# Bacterial carbonic anhydrase as a candidate vaccine target against *Helicobacter pylori*

**DOI:** 10.3389/fmicb.2025.1541387

**Published:** 2025-04-09

**Authors:** György M. Buzás

**Affiliations:** Ferencváros Health Centre, Department of Gastroenterology, Budapest, Hungary

**Keywords:** carbonic anhydrase, ethoxzolamide, *Helicobacter pylori*, messenger RNA, vaccine

## Introduction

The lack of an ideal eradication therapy prompted researchers to seek alternative ways to prevent and treat *H. pylori*-related diseases. Many vaccine types have been developed against *H. pylori*, including inactivated whole-cell vaccines, urease, outer membrane proteins, heat shock proteins, lipopolysaccharide, cytotoxin-associated gene A, flagellar sheath protein, DNA vaccines, recombinant *Salmonella typhimurium, Bacillus subtilis, Saccharomyces cerevisiae*, and measles virus vaccine), but carbonic anhydrase (CA) has been omitted (Zhang et al., 2022). Only a few made it to human trials; therefore, new areas of research must be explored. Although the worldwide prevalence of *H. pylori* decreased from 58.2 to 43.1% between 1980 and 2022 (Li J. et al., 2024), effective vaccination against *H. pylori* infection should be possible to prevent gastric cancer and other, less life-threatening conditions associated with this infection (Ilic and Ilic, 2022).

## Carbonic anhydrases in brief

Carbonic anhydrase (CA; EC 4.2.1.1) was discovered in 1933. Its role in gastric acid secretion was demonstrated in 1939. Acetazolamide, an enzyme inhibitor, was synthesized in 1950. CAs have a molecular mass of approximately 35,000–45,000 kDa and are highly conserved, with both structural homology and organ specificity. The spatial structure and protein folding, as well as the zinc-containing active center and mechanism of action, were characterized (Supuran, 2024). CA inhibitors were used in the 1980s to treat peptic ulcers, resulting in high healing rates (>90%) and low relapse rates (<10%). At that time, this was seen as the result of strong acid inhibition. Later, the low relapse rate was regarded as a possible effect of *H. pylori* eradication (Buzás and Supuran, 2016).

## Bacterial carbonic anhydrase

Bacterial *H. pylori* CA (*Hp*CA) has two forms: *Hp*CAά and *Hp*CAβ, which are located on the membrane, in periplasma and cytoplasm, respectively. The membrane-bound CA is either linked to the cell surface or enclosed in the outer membrane vesicles (Li Y. et al., 2024). The three-dimensional structure resembles that of human CAs; however, the amino acid sequence presents differences in the active catalytic site and other protein chain segments (Supuran and Camasso, 2017). Both *Hp*CAs are involved in the acid acclimation of *H. pylori*. *Hp*CA inhibitors, such as ethoxzolamide, kill bacteria *in vitro* at low concentrations, indicating that the enzyme is essential for bacterial survival (Supuran, 2024). Monoclonal antibodies against human and bacterial soluble and membrane-bound CAs were recently generated to identify enzymatic isoforms (Stravinskiene et al., 2019).

## Immunology of CAs

Autoantibodies against CA isoenzymes occur in patients with rheumatoid arthritis, systemic lupus erythematosus, polymyositis, systemic sclerosis, Sjögren's syndrome, autoimmune liver disease, diabetes mellitus, and endometriosis (D'Cruz et al., 1996; Liu et al., 2012). In genetically predisposed patients, *H. pylori* can cause autoimmune pancreatitis by mimicking molecular functions. *In silico* protein analysis showed homology between the pancreatic CA II isoform and *Hp*CA, with homologous segments containing the binding motif of an HLA molecule (Guarneri et al., 2005). Moreover, human CA IX can serve as a tumor marker in renal cell carcinoma, which can be identified by monoclonal antibodies. A recombinant heat shock protein and CA IX-based vaccine were even evaluated for targeting renal cell carcinoma (Combe et al., 2015). The association of *H. pylori* with immune thrombocytopenic purpura, Hashimoto's disease, rheumatoid arthritis, autoimmune hepatitis, and chronic urticaria is rather due to pro-inflammatory cytokines and virulence factors than to CA antibodies (Wang et al., 2023).

Recently, several attempts have been made to identify epitopes of *H. pylori* virulence factors. First, Chinese authors developed a multivalent epitope-based vaccine using specific antigens (urease, lipopolysaccharide 20, *Hp* adhesin A, and CagL). The specificity, immunogenicity, and antibody production were tested in BALB/mice, and the multiepitope vaccine proved to be more effective than the anti-urease vaccine (Guo et al., 2017). Another Chinese research group prepared antibodies to cytotoxin-associated gene A, vacuolating cytotoxin-associated gene A, and urease A and B genes (Du et al., 2023). An international group determined the crystal structure of *H. pylori* adhesin A, which plays an important role in cell adhesion of the bacterium and induces TNF-alpha production. The results could contribute to further vaccine preparation against this important virulence factor (Martini et al., 2024). A Bangladeshi research group identified outer-membrane proteins from *H. pylori* and examined them to identify cytotoxic and helper T lymphocytes as well as B cell epitopes, before developing a non-allergic, immunogenic vaccine. The non-toxic, soluble preparation binds to toll-like receptor 4. *In silico* testing and immune simulation revealed that it can able to initiate an immune response in humans. The authors suggest that it has the potential to induce robust immunity against *H. pylori* (Tamanna and Rahman, 2023). The Mexican authors used baculovirus carrying the Thp1 transgene coding for epitopes from urease B, CagL, Cag7, gamma-glutamyl transpeptidase, and CA, to produce a multiepitope recombinant baculovirus Th1 protein that was then inoculated in mice. A strong IgG response was obtained after intranasal, intragastric, intramuscular, and combined administration, which persisted in sera after 125 days, while IgA antibodies were found in feces after 82 days. Except for those using baculovirus, none of the above studies used CA as a target (Montiel-Martinez et al., 2023). Finally, an Iranian research group developed a multi-epitope vaccine using lipid nanoparticles that targeted five *H. pylori* proteins (urease, CagA, HopE, BabA, and SabA), but CA was omitted. The developed product was non-toxic and non-allergic, but further research is needed to establish its immunogenicity and safety (Jebali et al., 2024).

## Proposal and perspective

Knowing the role of human CA in acid secretion and *Hp*CA in acid acclimation of the bacterium, as well as the recent results in the field of *Hp*CA immunology and vaccinological research, I propose identifying specific *Hp*CA epitopes as single or multiple structures and generating specific antibodies to avoid cross-reaction with other CAs. It is noteworthy that antibodies against other *H. pylori* proteins (CagA, VacA, urease, GGT, heat-shock proteins, etc.) have not been developed into efficient human vaccines. After selecting the specific antibodies against bacterial CA, a new vaccine should be prepared, using the mRNA method as designed by Nobel laureate Katalin Karikó (Karikó et al., 2008; Pardi et al., 2020). CAs are vital for survival in all *H. pylori* strains; therefore, a vaccine targeting the enzyme can have an advantage over vaccines targeting other virulence factors that are not present in all strains and not essential for survival. After adequate laboratory, animal, and human testing, such preparations could be used as a vaccine against *H. pylori* infection, hopefully with more success than before. Some mRNA-based vaccines have already been developed against *Clostridioides difficile* (Alameh et al., 2024), *Listeria monocytogenes* (Mayer et al., 2022), and *Pseudomonas aeruginosa* (Wang et al., 2023). Why then must *H. pylori* be an exception? An effective vaccine can change the global epidemiology and clinical impact of *H. pylori* infection.
